# Comparative Analysis of Environmental Disinfection Methods: Hydrogen Peroxide Vaporization Versus Standard Disinfection Practices—An Experimental Study and Literature Review

**DOI:** 10.3390/jcm14113789

**Published:** 2025-05-28

**Authors:** Su Ha Han, Jung-Eun Yu, Seung Boo Yang, Young-Won Kwon, Minji Kim, Seong Jun Choi, Jung Wan Park

**Affiliations:** 1 College of Nursing, Soonchunhyang University, Cheonan 31151, Republic of Korea; jasmin720@hanmail.net; 2 Department of Internal Medicine, Division of Infectious Disease, Soonchunhyang University Hospital, Cheonan 31151, Republic of Korea; jungeunyu@hanmail.net (J.-E.Y.); kywon012@naver.com (Y.-W.K.); mming0825@gmail.com (M.K.); 3 Department of Radiology, Nowon Eulji University Hospital, Seoul 01830, Republic of Korea; ysbysb28@gmail.com; 4 Department of Otolaryngology-Head and Neck Surgery, Cheonan Hospital, College of Medicine, Soongchunhyang University, Cheonan 31151, Republic of Korea

**Keywords:** hydrogen peroxide vaporization, environmental disinfection, sodium hypochlorite

## Abstract

**Background/Objectives:** During the COVID-19 pandemic, the importance of disinfection and quarantine significantly increased, particularly in situations of staff shortages. Automated disinfection methods, such as hydrogen peroxide vaporization (HPV), are increasingly considered as alternatives to traditional manual disinfection. This study aimed to evaluate the efficacy of HPV compared to standard disinfection practices. **Methods:** Experiments were conducted at the Infectious Disease Clinical Research Simulation Center of Soonchunhyang University Hospital using Geobacillus stearothermophilus spores as biological indicators. The spores were inoculated on various hospital surfaces and allowed to dry for 120 min. Three disinfection methods were tested: (1) scrubbing with a disposable towel soaked in sodium hypochlorite; (2) placing sodium hypochlorite-soaked towels on the surface for one minute; and (3) HPV alone. Samples were collected post-disinfection and incubated at 55–60 °C. Bacterial cultures were assessed after 24, 48, and 168 h. **Results:** After 24 h of incubation, sterilization rates were 0% for the scrubbing method, 27% for sodium hypochlorite towels, 68% for HPV alone, and 95% for the combination of sodium hypochlorite and HPV. HPV alone demonstrated statistically greater efficacy compared to standard disinfection practices (*p* = 0.03). **Conclusions:** HPV alone may serve as a viable disinfection method in clinical environments, particularly during pandemics when staffing limitations hinder thorough manual cleaning. Further clinical trials are warranted to validate these findings and improve disinfection methods for challenging materials such as fabrics.

## 1. Introduction

With the coronavirus disease 2019 (COVID-19) pandemic persisting for over three years, disinfection and quarantine have become increasingly important [1]. These measures are essential for medical institutions responsible for isolating patients with infectious diseases, as they prevent transmission to other patients and ensure the safety of medical personnel. However, during the peak of the COVID-19 pandemic, most medical institutions faced staff shortages and severely increased workloads [2], leading to insufficient disinfection of hospital rooms with COVID-19 patients.

Recognizing these problems, numerous researchers and national policymakers have acknowledged the urgent need for automated disinfection of infectious disease wards [3]. Two primary methods have emerged as alternatives for automated disinfection: ultraviolet-C (UVC) disinfection and hydrogen peroxide (H_2_O_2_) vaporization (HPV). However, previous reports have shown that UVC disinfection may not be suitable as a terminal disinfection method for hospital rooms due to the presence of numerous hospital supplies and patient belongings, which hinder the penetration and effectiveness of UVC light [4,5]. On the other hand, HPV has only been used as an auxiliary method to date, usually after manual disinfection using sodium hypochlorite; thus, it is necessary to assess the potential of HPV as a terminal disinfection method [6,7,8]. Standard disinfection practices for environmental surfaces typically involve the use of sodium hypochlorite. The Centers for Disease Control and Prevention (CDC) recommends using a disposable towel soaked in sodium hypochlorite diluted to 1000 ppm, which is effective in disinfecting various surfaces. However, manual application can be labor-intensive and may miss hard-to-reach areas, highlighting the need for efficient disinfection methods.

Given these limitations, the challenges of thorough disinfection of hospital rooms using manual methods highlight the potential benefits of HPV as an automated solution. In this study, we aim to demonstrate that HPV is as effective as standard disinfection practices by employing laboratory comparisons of the effectiveness of H_2_O_2_ vaporization and traditional disinfection techniques. The study used an indicator of high-level disinfection or sterilization, which is different from general surface disinfection.

## 2. Materials and Methods

The study was conducted from July 26 to 27 in 2023 at the Infectious Disease Clinical Research Simulation Center of S University Hospital, City C. The simulation center had an area of 48.7 m^2^ and was designed to resemble a medical environment. The center was located in the basement of the building and had a rectangular floor plan with a single column in the center. Before starting the experiment, the room temperature was measured and found to be between 23 and 26 °C and the relative humidity between 70 and 80%. Since such high humidity can affect the effectiveness of hydrogen peroxide vaporization, the humidity was controlled using a dehumidifier before starting the vaporization process, and the experiment commenced at a relative humidity of 50 to 60%.

Three disinfection methods were compared: (1) scrubbing with a disposable towel soaked in sodium hypochlorite diluted to 1000 ppm for one minute and wiping with a clean wipe; (2) placing a disposable towel soaked in sodium hypochlorite diluted to 1000 ppm on the surface for one minute and wiping with a clean wipe; and (3) disinfection with hydrogen peroxide vaporization without any terminal cleaning.

The first two methods are based on standard disinfection practices recommended by the CDC. While these methods are not officially standardized, they have been widely implemented in healthcare settings. The third method involves the use of hydrogen peroxide vaporization, evaluated as a potential automated disinfection solution.

The room was thoroughly cleaned before each disinfection method was applied to ensure that the results were not influenced by residual contaminants from previous tests. Each disinfection method was repeated three times to ensure consistency and reliability in the results. The effectiveness of each method was evaluated by collecting surface samples from multiple predetermined locations within the simulation center before and after the disinfection process.

To control potential confounding variables, the same personnel performed all disinfection methods, and the same equipment and materials were used throughout the study. The hydrogen peroxide concentration and dispersion rate were also standardized and monitored to ensure uniform application during the vaporization process.

### 2.1. Setting and Infected Assay

The surfaces of various items (10 in total) in the center, including tables, curtains, mattresses on stretcher carts, medical trays and patient monitoring devices, were inoculated with the standard strain for the experiment (Spore Suspension *Geobacillus stearothermophilus* 7953, MesaLabs, Lakewood, CO, USA). To inoculate the same amount of strain on the environmental surface, the suspension (STEAM 7953, MesaLabs), which was 10^9^ CFU/10 mL, was quantified to 10^7^ CFU/0.1 mL by dispensing 0.1 mL into a 1 ml sterile disposable syringe and then spread evenly over a 5 cm^2^ area of the environmental surface. Each disinfection method was independently replicated three times on ten different surfaces, resulting in a total of 30 observations per method. To evaluate the effectiveness, the environmental surface inoculated with the experimental strain was dried for two hours and then fumigated with hydrogen peroxide, and a sterile walking robot equipped with a FAN was operated to ensure that the hydrogen peroxide vapor evenly reached the end of the experimental space. Chemical Indicators (CI, RENO Chemical Indicator Strip, Renosem, Incheon, Republic of Korea) were placed at various places to check the diffusion of hydrogen peroxide vapor in the space.

After hydrogen peroxide vaporization was completed, the examiner donned sterile gloves, a disposable gown and a mask, and the environmental surfaces that had been inoculated with the strain were swabbed with sterile swabs moistened with sterile distilled water and inoculated into Trytic Soy Broth medium (Trytic Soy Broth with Bromocresol Purple TSB-BP16, MesaLabs). Two control groups were prepared: a negative control of TSB medium with the lid open to the air and a positive control of TSB medium inoculated with the experimental standard strain only.

The process of inoculating the strain and collecting environmental surface samples was performed by one infection control specialist and one researcher with more than 10 years of infection control experience. The inoculated medium was placed in an incubator at 55 to 60 °C for 48 h. The sterilization of the incubation was determined successful if the color of the culture medium remained purple and unsuccessful if it turned yellow. Chemical Indicators (CI) indicated the presence of hydrogen peroxide vapor when the color changes from purple to pink.

### 2.2. Sodium Hypochlorite Disinfection

In accordance with environmental disinfection methods recommended by the CDC, the surfaces of a total of 10 different items inoculated with the experimental strain were subjected to a contact time of at least one minute with sodium hypochlorite disinfectant diluted to 1000 ppm [7]. For disinfecting wipes, contact time included wet times, wiping time, and undisturbed time [9]. The disinfectant must be exposed to the surface for a sufficient amount of time before it can be determined that the disinfectant is effective enough to kill the contaminants on the surface.

First, a disposable towel soaked in sodium hypochlorite diluted to 1000 ppm and folded three times was used to wipe off the first visible organic matter. The towel was flipped every 30 s to switch to its new side and wiping of the surface was continued longitudinally back and forth for a total of one minute. The surface was then wiped longitudinally from front to back with a clean wipe.

Secondly, a disposable towel soaked in sodium hypochlorite diluted to 1000 ppm was placed on the inoculated surface for one minute and the surface wiped vertically from front to back. After switching to its new side, the surface was wiped again from front to back; the surface was later wiped vertically from front to back with a clean wipe.

### 2.3. Hydrogen Peroxide Vaporization

Following hydrogen peroxide vaporization protocols [7,8], bed mattresses were hung at an angle to the bed frame to facilitate the penetration of the sprayed solution, and all drawers of medical carts and side tables were left open. To prevent the sprayed solution from entering other spaces through the air conditioning, the air conditioning vents, ceiling and doorway gaps were sealed with tape, and no outside access was allowed during the experiment. After the hydrogen peroxide vaporization was completed, the hydrogen peroxide vapor was removed by a scrubber, and the tape was removed and replaced when the hydrogen peroxide concentration was below 1 ppm. Hydrogen peroxide vaporization was performed in three stages using 35% hydrogen peroxide: injection (60 min), dwell (30 min), and aeration (60 min), with a total duration of 2.5 h. During the hydrogen peroxide vaporization process, hydrogen peroxide concentration and relative humidity were measured at several locations.

### 2.4. Data Sources and Searches

This systematic review and meta-analysis involved a search of relevant articles in the PubMed, Ovid, Embase, Cochrane, and Web of Science databases from their inception to December 2022. The search queries were formulated based on PICO (patient-intervention-comparison-outcome) components using Boolean operators. Specifically, the main search terms included “hydrogen peroxide” in combination with either “disinfection” or “decontamination” and were extended based on Medical Subject Headings, entry terms, and related terms. We included only published studies that reported on the effectiveness of airborne hydrogen peroxide disinfection for eradicating bacterial contamination in hospital settings. Specifically, we selected studies focused on naturally dispersed pathogens in hospital environments, excluding experimental inoculation. Additionally, we included a study that evaluated disinfection by HPV as an infection control measure applied in an actual clinical environment.

## 3. Results

### 3.1. Analyze the Effectiveness of Disinfection

In sterilization testing, the “scrub for one minute and wipe” method with sodium hypochlorite diluted to 1000 ppm resulted in a 0% sterilization rate as bacteria were detected on all 10 environmental surface samples. The “leave the towel for one minute and wipe” method confirmed the killing of *Geobacillus stearothermophilus* on 3 out of 10 environmental surface samples (30%). The bacteria were not killed on the other surfaces, including stainless steel and plastic medical carts, the mattress of an embossed artificial stretch cart, the mattress of an operating table, a polyethylene sphygmomanometer cuff, plastic patient monitor handles, and polyester curtains (Table 1).

Hydrogen peroxide vaporization killed *Geobacillus stearothermophilus* on 6 out of 10 environmental surface samples (60%), resulting in a 7 log10 reduction. The bacteria remained viable on an embossed leatherette operating table mattress, a polyethylene sphygmomanometer cuff, an acrylic board, and plastic patient monitor handles (Table 1).

### 3.2. Distribution and Scrubbing of Hydrogen Peroxide

**Figure 1:**
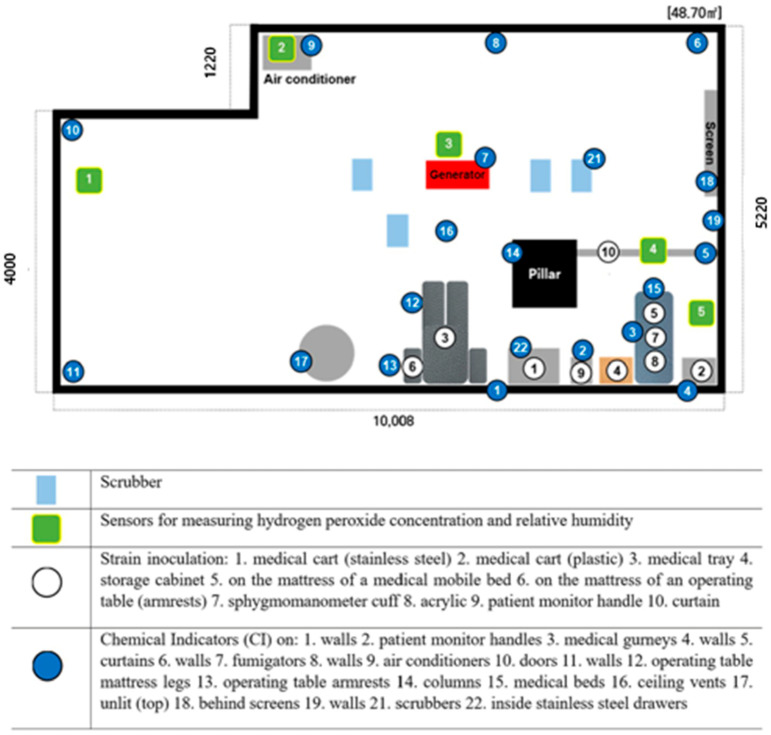
Diagrams of pickup, furniture and strain inoculation locations and Chemical Indicators (CIs) in the lab space.

The relative humidity at the start of the hydrogen peroxide vaporization application was 52 to 60%, and the peak humidity during the vaporization process was 83 to 85%. The hydrogen peroxide concentration was measured to be as high as 240 to 270 ppm next to the fumigator and above the air conditioner in the center of the room, less than 200 ppm on the floor in front of the doorway, and 200 to 210 ppm on the floor and curtain rods inside the test space (Figure 1). The environment and conditions of our experiments are described in the Supplementary Table S1. At the time of the experiment, the humidity was between 57 and 83%, and the maximum concentration of H_2_O_2_ was 263 ppm.

The Chemical Indicators (CIs) attached to check the diffusion of hydrogen peroxide vapor into the room did not turn pink behind the walls and screens in the innermost parts of the room, confirming that hydrogen peroxide vapor did not fully diffuse through.

## 4. Discussion

Our analysis confirmed that HPV disinfection is not inferior to standard disinfection practices in terms of effectiveness. This supports our hypothesis that HPV alone can provide a certain level of disinfection effect, even without performing standard disinfection practices.

During the COVID-19 pandemic, most healthcare organizations focused on stopping the spread of the virus [10]. As a result, epidemic prevention authorities required medical institutions to strictly adhere to standards of disinfection practices and sterilization [11,12]. However, the COVID-19 pandemic has exposed the vulnerability of our healthcare system, particularly the shortage of healthcare workers, which prevents healthcare facilities from maintaining adequate disinfection [13,14]. Additionally, the need to wear level D protective gears in hospital rooms with COVID-19 patients made healthcare workers reluctant to perform complex disinfection. As a result, health authorities have explored automated disinfection methods to reduce the need for medical staff intervention. The most popular methods of automated disinfection are UV disinfection and HPV [5].

HPV involves using vaporized hydrogen peroxide to deactivate or kill microorganisms on surfaces and in the air. The H_2_O_2_ vapor is generated by special machines called hydrogen peroxide vaporizers. It diffuses into the air, reaches all surfaces and areas in a room, including those that are difficult to inspect and clean with traditional disinfection methods, such as the undersides of counters, tabletops and equipment, and kills microorganisms on contact. Therefore, HPV has been proven to be an effective method for disinfecting hospital environments [6].

HPV offers several benefits. It can disinfect a room relatively quickly. Previous studies have shown that it can achieve a 6-log reduction in bacterial and viral pathogens within as little as 30 min. Moreover, H_2_O_2_ is a non-toxic disinfectant and its vaporization does not leave harmful residues or byproducts, ensuring safe usage in hospital environments. Additionally, unlike other disinfection methods, such as bleaching or UV light, HPV does not damage surfaces or equipment. It is compatible with sensitive materials like electronics, fabrics and plastics. Lastly, HPV is cost-effective compared to other disinfection methods, requiring fewer resources and personnel, and can be completed in a short time frame [15].

However, despite its benefits, HPV has some drawbacks that hinder its use as a routine disinfection method. Firstly, although HPV is effective against many bacterial and viral pathogens, it may not be equally effective against all types of microorganisms. Some studies have suggested that it may be less effective against certain spore-forming bacteria like *Clostridium difficile*. Additionally, although H_2_O_2_ is considered safe in general, its vaporization can pose safety concerns arising from the potential irritation of the eyes, respiratory tracts and skin, as well as the flammability at high concentrations. Moreover, HPV requires specialized equipment, which can be expensive. Its maintenance costs and the need for trained personnel to operate the equipment may contribute to the overall expenses. Finally, the generation of large amounts of waste, including used cartridges and packaging materials, can have a negative impact on the environment if not properly managed. As a result, most hospital environmental disinfection guidelines recommended using HPV after standard disinfection practices. However, as the pandemic required an automated disinfection method that minimizes human intervention, our research investigated the effectiveness of HPV disinfection alone, without involving standard disinfection practices [16,17,18].

Our results demonstrate that HPV is non-inferior to standard disinfection practices, albeit in a limited setting. Additionally, even when HPV disinfection was used without standard disinfection, it exhibited notable effects in disinfecting the environment and preventing the spread of infectious diseases. The HPV used in this study is a high-level disinfection method, equivalent to sterilization, and is not the same as general surface disinfection.

To further substantiate our experimental findings, we conducted additional analysis through a literature review. Among the studies that used HPV as a disinfection method in clinical settings, we selected 10 papers that demonstrated the effectiveness of disinfection through environmental samples and conducted a meta-analysis (Table 2) [19,20,21,22,23,24,25,26,27,28]. Among the 10 included studies, four of them analyzed the efficacy of HPV alone without applying standard disinfection practices. In the other six studies, HPV was applied after standard disinfection practices, following typical terminal cleaning practices. Among the six studies, one study analyzed environmental samples for three pathogens from different sites, necessitating separate statistical analyses. Two studies involved a study design that allowed a direct comparison of the efficacy of HPV with standard disinfection. Therefore, we analyzed the non-inferiority of HPV by conducting a step-by-step comparison of HPV with standard disinfection and evaluating their efficacies.

**Figure 2:**
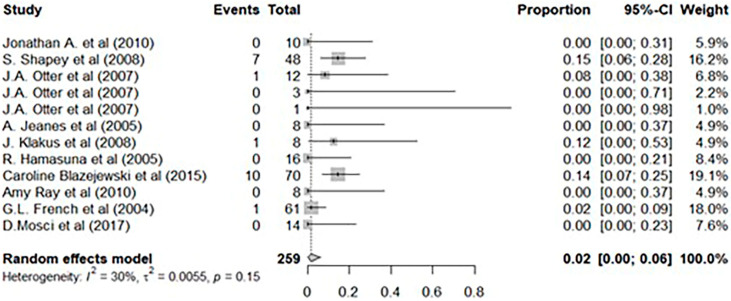
Differences between consensus estimates of the efficacy of hydrogen peroxide vaporization [19,20,21,22,23,24,25,26,27,28].

**Figure 3:**
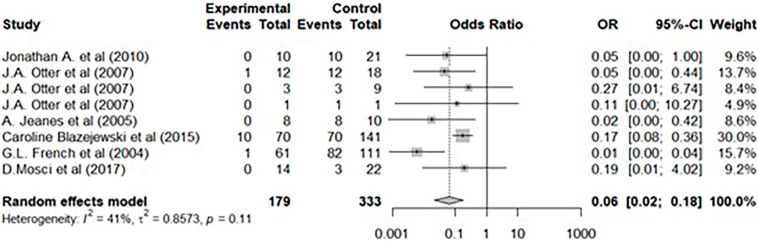
Differences in efficacy between standard disinfection and hydrogen peroxide vaporization [19,21,22,25,27,28].

**Figure 4:**
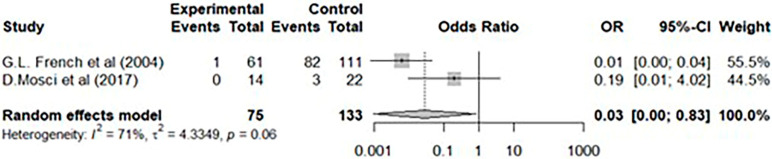
Head-to-head analysis of hydrogen peroxide vaporization versus standard disinfection [27,28].

The heterogeneity assessment of these 10 selected studies indicated a small heterogeneity with an I^2^ value of 30.46% and no statistically significant heterogeneity (*p* > 0.1). In an analysis of the number of positive environmental swab samples before and after applying HPV, a random-effects model confirmed a significant effect with an odds ratio of 0.2 (95% CI: 0.00–0.06) (Figure 2). A random-effects model was employed to further estimate the number of positive environmental samples before and after standard disinfection practices versus those before and after HPV. The odds ratio was 0.06 (95% CI: 0.02–0.18), indicating that, compared with standard disinfection practices, HPV significantly reduced the incidence of positive environmental samples (Figure 3). A comparison was conducted between two studies that used different disinfection methods for various rooms. The experimental group measured the number of positive environmental swab samples before and after applying HPV, whereas the control group measured the number of positive environmental swab samples before and after standard disinfection practices. The odds ratios were 0.01 and 0.19 (95% CI: 0.00–0.04 and 0.01–4.02, respectively); the overall odds ratio estimated from the random-effects model was 0.03 (95% CI: 0.001–0.826), indicating that, compared with standard disinfection practices, HPV significantly reduced the incidence of positive samples (Figure 4).

However, this meta-analysis should be interpreted with caution due to the small number of studies sampled and the lack of study designs that directly compared HPV to standard disinfection.

Our study has the following limitations; First, our experiments were conducted in a medical device clinical trial center that mimics a medical setting, so we were unable to test disinfection in the presence of multiple body fluids, such as blood, and therefore disinfection may not be as effective in real-world situations. The experiment was not conducted in a real medical institution, so we cannot exclude the possibility that the effectiveness of disinfection may be slightly different in actual clinical practice. Second, since the test was conducted using *G. stearothermophilus*, it is safe to assume that the disinfection of most bacteria is theoretically proven, but the effectiveness of the disinfection of the actual causative agent has not been proven with certainty. Third, multiple samples per site would have added statistical power to the evidence, which was lacking.

In conclusion, our study suggests that the most ideal environmental disinfection strategy involves applying HPV as a method of terminal cleaning after performing standard disinfection practices, as HPV can achieve significant reductions in various pathogens. Our analysis proves HPV as a reliable alternative when standard disinfection practices are challenging to implement due to staff shortage or high contamination risks. We have further demonstrated that environmental disinfection with HPV alone is not inferior to standard disinfection practices. Therefore, in situations like the COVID-19 pandemic, in which extreme understaffing or exposure to pathogens in healthcare workers is prevalent, HPV alone may be a viable option for environmental disinfection. Despite its benefits, HPV has its limitations. It may not be equally effective against all types of microorganisms, such as certain spore-forming bacteria like *Clostridioides difficile*. Additionally, the use of HPV involves certain safety concerns and requires specialized equipment, which is costly and necessitates trained personnel for operation. Although routine use of HPV for terminal cleaning may not be feasible due to such constraints, it can be particularly useful in high-risk and resource-limited situations.

Additionally, while durable materials that can withstand rigorous cleaning protocols are recommended in healthcare settings, the disinfection of fabric-based items should also be considered and improved to ensure comprehensive infection control.

Future studies should focus on clinical trials with more sophisticated designs to further validate these findings. Additionally, methods to enhance the effectiveness of environmental disinfection on various materials such as fabrics, which are traditionally more challenging to disinfect, require further research. Addressing these challenges will be crucial for improving disinfection in healthcare institutions under resource-limited settings.

## Figures and Tables

**Table 1:** Effectiveness of disinfection methods in killing *Geobacillus stearothermophilus*.

No.	Investigation Subject	Material	Material (Picture)	Incubation Results		
				Wiping for 1 min *	Put on Surface for 1 min ^†^	Hydrogen Peroxide Vaporization
1	Medical cart	stainless steel	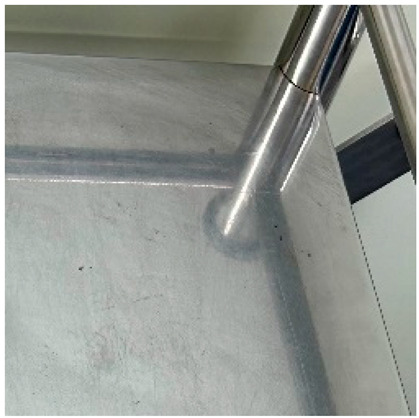	positive	positive	negative
2	Medical cart	plastic	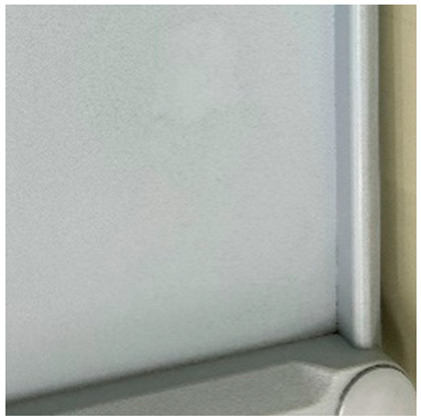	positive	positive	negative
3	Medical tray	stainless steel	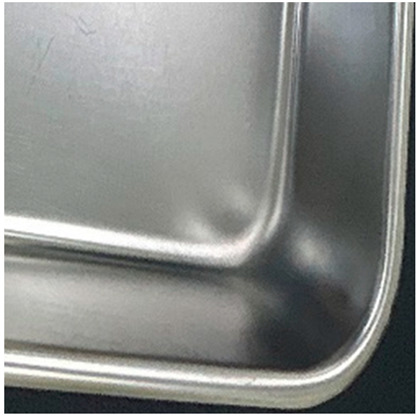	positive	negative	negative
4	The cabinet	MDF (Medium Density Fiberboard)	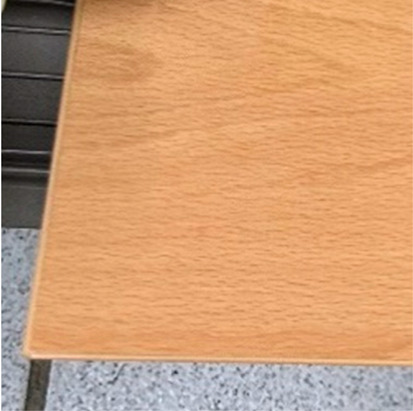	positive	negative	negative
5	Stretcher Car Mattress	artificial leather	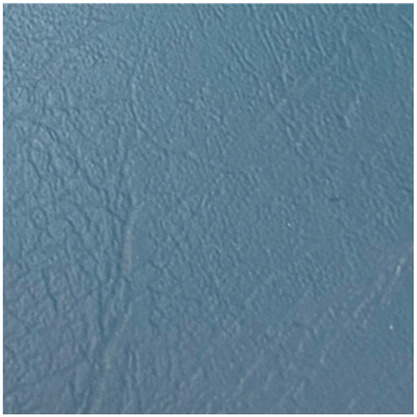	positive	positive	negative
6	Operating table mattress	artificial leather	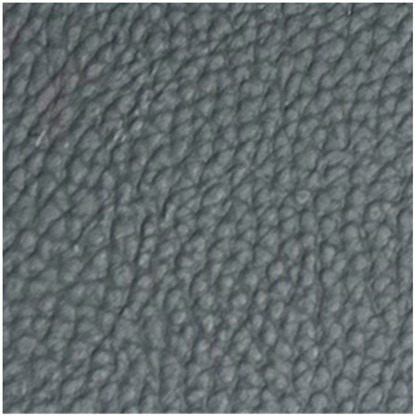	positive	positive	positive
7	Blood pressure cuff	polyurethane (Waterproof)	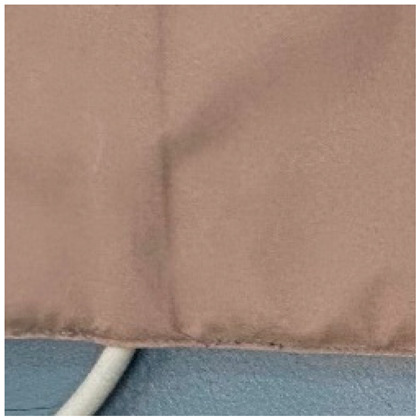	positive	positive	positive
8	Acrylic board	acryl	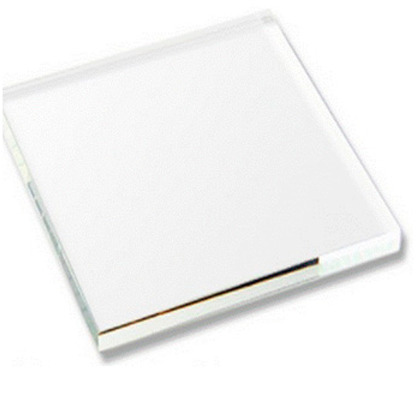	positive	negative	positive
9	Handle of patient monitoring device	plastic	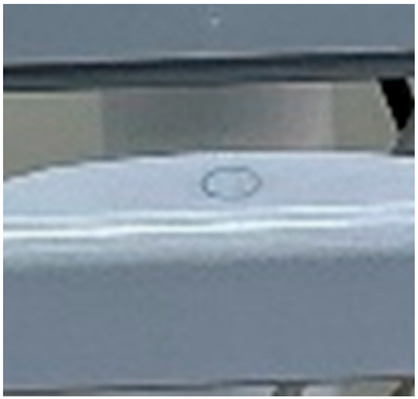	positive	positive	positive
10	Curtain	polyester	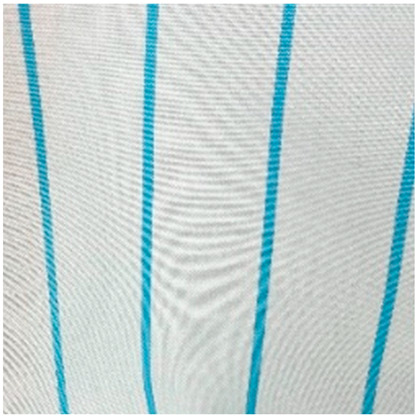	positive	positive	negative
Control	Negative control ^‡^	-	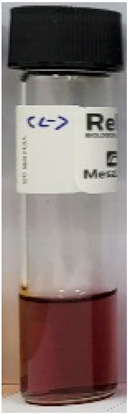	negative	negative	negative
	Positive control ^§^	-	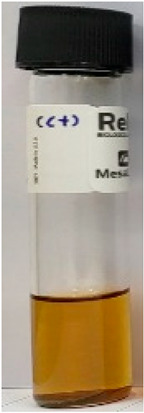	positive	positive	positive

* The surface was wiped for one minute with a disposable towel soaked in sodium hypochlorite diluted to 1000 ppm. ^†^ A disposable towel soaked in sodium hypochlorite diluted to 1000 ppm was placed on the surface for one minute. ^‡^ Trytic Soy Broth medium with cover left open to air. ^§^ Trytic Soy Broth medium inoculated with only experimental standard strains.

**Table 2:** Comparison of the number of environmental samples taken before and after disinfection in each study.

Article	Author	Target Pathogen	Whether Standard Disinfection Practice Was Applied	Number of Environmental Samples Acquired	Number of Positive Samples Before Standard Disinfection Practice	Number of Positive Samples After Standard Disinfection Practice	Number of Samples That Tested Positive Before HPV Was Performed	Number of Samples That Tested Positive After HPV Was Performed
Am. J. Infect Control. (2010) [19]	A. Jonathan et al.	MDR-GNR	Yes	63	21	10	10	0
J. Hosp. Infect. (2008) [20]	S. Shapey et al.	*Clostridioides difficile*	No	203	-	-	48	7
J. Hosp. Infect. (2007) [21]	J.A. Otter et al.	MRSA	Yes	30	18	12	12	1
		GNR	Yes	30	9	3	3	0
		VRE	Yes	15	1	1	1	0
J. Hosp. Infect. (2005) [22]	A. Jeanes et al.	MRSA	Yes	50	10	8	8	0
J. Hosp. Infect. (2008) [23]	J. Klakus et al.	MRSA	No	29	-	-	8	1
J. Hosp. Infect. (2005) [24]	R. Hamasuna et al.	All cultured	No	42	-	-	16	0
Crit. Care. (2015) [25]	Caroline Blazejewski et al.	MDRO	Yes	182	141	70	70	10
ICHE (2010) [26]	Amy Ray et al.	MRAB	No	93	-	-	8	0
J. Hosp. Infect. (2004) [27]	G.L. French et al.	MRSA	Yes	124	111	82	61	1
J. Hosp. Infect. (2017) [28]	D. Mosci et al.	*Clostridioides difficile*	Yes	112	22	3	14	0

HPV, hydrogen peroxide vaporization; MDR-GNR, Multidrug-resistant Gram Negative Rods; MRSA, Methicillin-resistant *Staphylococcus aureus*; VRE, Vancomycin-resistant Enterococcus; MDRO, Multidrug-resistant organism; MRAB, Multidrug-resistant *Acinetobacter baumannii*.

## Data Availability

The data presented in this study are available on request from the corresponding author.

## Supplementary Materials

The following supporting information can be downloaded at: https://www.mdpi.com/article/10.3390/jcm14113789/s1, Table S1: The experimental conditions and the experimental environment.
