# Supplementary material for: Comparative Analysis of Environmental Disinfection Methods: Hydrogen Peroxide Vaporization Versus Standard Disinfection Practices—An Experimental Study and Literature Review
Source: J Clin Med. 2025 May 28;14(11):3789. doi: 10.3390/jcm14113789 (PMC12155998; doi:10.3390/jcm14113789)
Supplement: Supplementary file 1 [file jcm-14-03789-s001.zip › jcm-3625070-supplementary.pdf]

Supplementary Table S1. The experimental conditions and the experimental environment.

**Surface inoculate**	the strain 10^9CFU/10mL in 1 vial, so 10^7CFU/0.1mL (10^7CFU per 0.1mL) ⇒ dispense 0.1mL (10^7CFU) on one spot
**Strain drying**	total 120 minutes (approx. 2 hours)
**Vaporization Disinfection**	15:16 Start spraying at 58-60% humidity ⇒ 18:13 End disinfection (total time 2 hours 57 minutes)<br>Spraying approx. 1 hour 20 minutes, rest 30 minutes, disintegration approx. 1 hour
**Collection and culture**	Collect the surface-applied bacteria with a sterile swab moistened with sterile distilled water → Inoculate the collected bacteria into the prepared culture medium and transfer them to a broth.<br>→ Incubate in a 55~60°C incubator for 7 days
**Strain inoculation area**	5*5 cm area, spread wider than the first one
**Maximum H_2O_2 concentration**	263 ppm
**Disinfectant spray rate**	13 cc/min
**Usage per volume / total usage**	8 cc / 1080 cc
**Relative humidity**	57~86.3 %RH
